# Multifaceted Effects of Kinase Inhibitors on Pancreatic Cancer Cells Reveals Pivotal Entities with Therapeutic Implications

**DOI:** 10.3390/biomedicines11061716

**Published:** 2023-06-15

**Authors:** Yoo Na Kim, Ketki Patil, Jeonghwa Ma, Griffin A. Dufek, S. Balakrishna Pai

**Affiliations:** Wallace H. Coulter Department of Biomedical Engineering, Georgia Institute of Technology and Emory University, 313 Ferst Drive, Atlanta, GA 30332, USA; ynkim4@uci.edu (Y.N.K.); kpatil7@mail.gatech.edu (K.P.); jeonghwa9919@yonsei.ac.kr (J.M.); grifffindufek@gmail.com (G.A.D.)

**Keywords:** pancreatic cancer, PANC-1, BxPC-3, Aurora kinase inhibitor, EGFR kinase inhibitor, apoptosis, ROS, proteomics, PD-L1, vimentin, lactoferrin, inhibitors of metastasis, protein–protein interaction

## Abstract

Pancreatic cancer is one of the most aggressive forms of cancer and is the seventh leading cause of cancer deaths worldwide. Pancreatic ductal adenocarcinoma (PDAC) accounts for over 90% of pancreatic cancers. Most pancreatic cancers are recalcitrant to radiation, chemotherapy, and immunotherapy, highlighting the urgent need for novel treatment options for this deadly disease. To this end, we screened a library of kinase inhibitors in the PDAC cell lines PANC-1 and BxPC-3 and identified two highly potent molecules: Aurora kinase inhibitor AT 9283 (AT) and EGFR kinase inhibitor WZ 3146 (WZ). Both AT and WZ exhibited a dose-dependent inhibition of viability in both cell lines. Thus, we conducted an in-depth multilevel (cellular, molecular, and proteomic) analysis with AT and WZ in PANC-1 cells, which harbor KRAS mutation and exhibit quasimesenchymal properties representing pancreatic cancer cells as having intrinsic chemoresistance and the potential for differential response to therapy. Elucidation of the molecular mechanism of action of AT and WZ revealed an impact on the programmed cell death pathway with an increase in apoptotic, multicaspase, and caspase 3/7 positive cells. Additionally, the key survival molecule Bcl-2 was impacted. Moreover, cell cycle arrest was observed with both kinase inhibitors. Additionally, an increase in superoxide radicals was observed in the AT-treated group. Importantly, proteomic profiling revealed differentially regulated key entities with multifaceted effects, which could have a deleterious impact on PDAC. These findings suggest potential targets for efficacious treatment, including a possible increase in the efficacy of immunotherapy using PD-L1 antibody due to the upregulation of lactoferrin and radixin. Furthermore, combination therapy outcomes with gemcitabine/platinum drugs may also be more effective due to an increase in the NADH dehydrogenase complex. Notably, protein–protein interaction analysis (STRING) revealed possible enrichment of reactome pathway entities. Additionally, novel therapy options, such as vimentin-antibody--drug conjugates, could be explored. Therefore, future studies with the two kinases as monotherapy/combination therapy are warranted.

## 1. Introduction

Pancreatic cancer generally refers to pancreatic ductal adenocarcinoma (PDAC) as it accounts for 90% of pancreatic cancers identified. This malady is aggressive in nature, with a 5-year survival rate below 10% [1], and it is the seventh leading cause of cancer death worldwide and third in the United States [2,3]. It is estimated that PDAC could cause even higher cancer deaths by the year 2025 in the United States [4]. Long-term survival for patients with pancreatic cancer is rare [5]. In human PDAC, nearly all *ras* mutations are localized in the KRAS [6]. Apart from mutations, due to tumor and stromal interactions, novel therapeutic strategies, such as combination therapies, are needed for pancreatic cancer [7].

Pancreatic cancers are known to be recalcitrant to radiation therapy as well as chemotherapy. Currently, the treatment for patients with resectable and advanced PDAC consists of a combination therapy with mFOLFIRINOX (modified 5-fluorouracil, oxaliplatin, irinotecan, and leucovorin) [8,9]. Immunotherapy, which is harnessed by neutralizing antibodies to checkpoint entities such as PD-1 and its ligand PD-L1, has been successful for various cancers; however, limited efficacy has been observed in pancreatic cancer [10,11] and also for anti-CTLA-4 [12]. Notably, personalized RNA vaccines (neoantigens) have been reported recently to stimulate T cells in pancreatic cancer, with 50% of patients exhibiting a delayed recurrence of the cancer [13].

Many efforts and clinical trials have been conducted in a quest for novel therapies for this highly aggressive cancer, including targeting critical signaling pathways controlled by kinases [14]. Among the kinases, EGFR kinase is a promising target due to the overexpression of EGFR in pancreatic cancers; in particular, a treatment strategy mediated by nanoparticles targeting the EGFR ligand has been reported for pancreatic cancers [15,16]. Notably, the expression of epidermal growth factor (EGF) and its receptor (EGFR) as prognostic factors are reported in certain pancreatic cancer patients [17]. Further, clinical trials of the EGFR inhibitor, Erlotinib have shown modest success in combination with gemcitabine for patients with EGFR mutations [18]. Among other kinases, Aurora kinase A has been reported to be overexpressed in pancreatic cancer and is also reported to play a role in causing dysplasia in pancreatic ducts [19,20,21]. Moreover, Aurora kinase A has been reported as a downstream target in the MAPK1/ERK2 signaling pathway in pancreatic cancer [22]. Therefore, targeting Aurora kinases in gastrointestinal cancers could be considered as an attractive strategy [23]. In particular, PANC-1 could be inhibited by Autora Kinase inhibitors, as this is suggested as a potential strategy for pancreatic cancers harboring KRAS mutations [24].

The goal of the present study was to identify potential pivotal molecules with implications for novel and efficacious treatment options for pancreatic cancer, a disease with limited treatments available and notorious for its aggressive nature. We performed an in-depth multilevel analysis with two potent kinase inhibitors, Aurora kinase inhibitor AT 9283 (AT) and EGFR kinase inhibitor WZ 3146 (WZ), in the PANC-1 pancreatic adenocarcinoma cell line, which harbors the predominant KRAS mutation G12D [25] and exhibits a quasimesenchymal phenotype [26]. This subtype represents pancreatic cancer cells with intrinsic chemoresistance, which might lead to differential responses to therapy. Moreover, we explored the mechanism of action of both the kinase inhibitors in this cell line to delineate the entities and pathways affected by the kinase inhibitors, which could have therapeutic implications.

## 2. Materials and Methods

### 2.1. Cell Lines and Materials

Kinase inhibitor library L1200 was purchased from Selleck Chemicals (Houston, TX, USA). AT 9283, abbreviated as AT, and WZ 3146, abbreviated as WZ in this manuscript, were identified as potent inhibitors from the kinase inhibitor library. A stock solution of 10 mM concentration of each compound was made in DMSO. For flow cytometry, Luminex Muse Cell analyzer and Muse assay kits were bought from EMD Millipore (Burlington, MA, USA).

Human pancreatic adenocarcinoma cell lines PANC-1 (CRL-1469) and BxPC-3 (CRL-1687) were purchased from ATCC (Manassas, VA, USA). The PANC-1 cells were grown in DMEM medium supplemented with 10% fetal bovine serum, 1% Penicillin-Streptomycin, and 2 mM L-glutamine. Cultures were incubated at 37 °C in an atmosphere of 5% CO_2_. BxPC-3 cells were grown under similar conditions as PANC-1 except that RPMI medium was used instead of DMEM.

### 2.2. Library Screening and Toxicity Assay

PANC-1 cells were plated in a 96-well plate at a density of 5000 cells per well in the culture medium. After 24 h, cells were treated with various concentrations of inhibitors ranging from 0.1 µM to 10 µM for the initial screening experiment. The selected potent kinase inhibitors, AT and WZ, were further assessed for impact on the viability of PANC-1 cells at various concentrations ranging from 0.05 µM to 20 µM. Cells were incubated for 72 h at 37 °C in an atmosphere of 5% CO_2_. The assays were carried out in triplicates. Cell Counting Kit-8 assay from Bimake (Houston, TX, USA) was used to assess the cell toxicity. The media was aspirated, and 200 µL of 10% CCK-8 solution in the complete growth medium was added. Absorbance was measured at 450 nm using an Infinite 200Pro plate reader (Tecan, Männedorf, Switzerland) after 1.5 h of incubation at 37 °C. The viability of cells was expressed after normalizing the treatment group absorbance values to that of the control group. Using Prism software, version 7.0c (GraphPad, Boston, MA, USA), graphs were plotted and IC_50_ was determined. Three independent experiments were performed for both screening and viability assays for AT and WZ. Identical screening and viability experiments with similar repetitions were conducted with the BxPC-3 cell line. For all mechanistic studies, PANC-1 cells were treated with AT at 8 µM and WZ at 5 µM (above IC_50_ concentration).

### 2.3. Flow Cytometry Assays

PANC-1 cells were plated in 12-well plates at 62,500 cells per well and after 24 h cells were left either untreated or treated in triplicate for 72 h with 8 µM of AT or 5 µM of WZ. After 72 h of incubation, media were collected, and then adhered cells were trypsinized and pooled together from triplicate treatments before proceeding with Luminex Muse assay kit protocol as per manufacturer’s instructions (Luminex Corporation, Austin, TX, USA). The following Luminex Muse assay kits were used: Oxidative Stress kit (MCH100111), Annexin V and dead cell kit (MCH100105), MultiCaspase kit (MCH100109), Caspase 3/7 (MCH100108), Bcl-2 activation dual detection kit (MCH200105), and Cell cycle kit (MCH100106).

### 2.4. Proteomic Analysis

Proteomic analysis was performed by 2D DIGE and mass spectrometry, which was conducted by Applied Biomics Inc. (Hayward, CA, USA) employing previously published methodologies [27]. PANC-1 cells were treated individually with 8 µM AT or 5 µM WZ. Control cultures (no treatment) were maintained in parallel. Cells from control samples and treated samples were collected, washed with 1×PBS, and then stored at −80 °C prior to sending the samples to Applied Biomics Inc. (Hayward, CA, USA) on dry ice for proteomic analysis. The protocol performed was as described previously [27,28,29]. STRING database version 11.5 was used to perform functional enrichment analysis and to generate protein–protein interaction networks [30].

### 2.5. Statistical Analysis

One-way ANOVA with Dunnett’s multiple comparison test was used for CCK-8 assay and histogram representation of flow cytometric assays with alpha set to 0.05. The *p*-values are represented in the legends for the figures.

## 3. Results

### 3.1. Identification of Molecules Impacting the Viability of PANC-1 and BxPC-3 Cells by Screening a Library of Kinase Inhibitors

**Figure 1:**
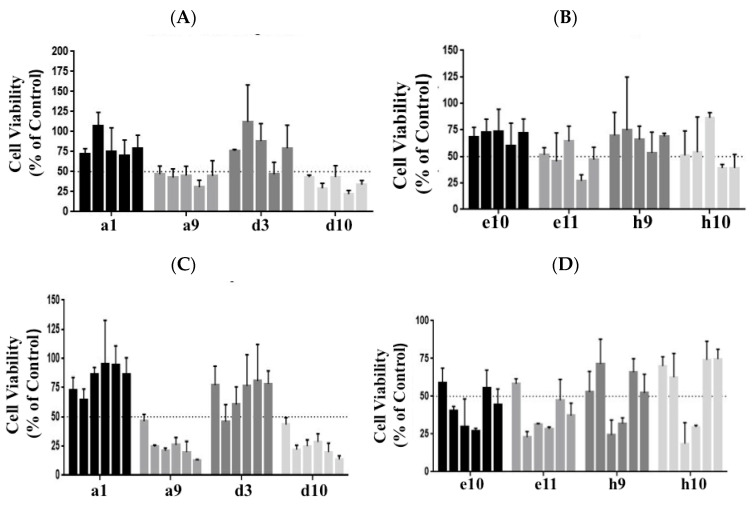
A set of compounds from the library of kinase inhibitors was tested at 0.1, 0.5, 1, 5, and 10 µM concentrations for their impact on the inhibition of PANC-1 cell viability. Cells were plated at a cell density of 5000 cell/well and incubated for 72 h. Cell viability was monitored using the CCK 8 assay. (**A**) Compounds labeled a1, a9, d3, and d10 from the library were assessed. (**B**) Compounds e10, e11, h9, and h10 were the next set assayed. Similar screening assays were performed for the cell line BxPC-3, (**C**,**D**), which ranged from 0.05 to 10 µM of the kinase inhibitors. The kinase inhibitors labeled a9 and d10 exhibited an impact on cell viability in both the cell lines and was identified as an Aurora kinase inhibitor, abbreviated AT, and the EGFR kinase inhibitor, abbreviated WZ, respectively. The bars represent cell viability at various concentrations. In (**A**,**B**) concentrations from left to right are 0.1 µM, 0.5 µM, 1 µM, 5 µM, and 10 µM. In (**C**,**D**), the concentrations from left to right are 0.05 µM, 0.1 µM, 0.5 µM, 1 µM, 5 µM, and 10 µM. Details of the compounds screened and their targets are as follows: **a1**: Linifanib (ABT-869)-CSF-1R, PDGFR, VEGFR; **a9**: AT9283-Aurora Kinase, Bcr-Abl, JAK; **d3**: Pazopanib HCl (GW786034 HCl)-c-Kit, PDGFR,VEGFR; **d10**: WZ3146-EGFR; **e10**: CYC116-Aurora Kinase, VEGFR; **e11**: YM201636-PI3K; **h9**: SNS-314 Mesylate-Aurora Kinase; **h10**: Regorafenib (BAY 73-4506)-c-RET,VEGFR.

A number of molecules were tested in the *PANC-1* and *BxPC-3* cell lines at various concentrations (ranging from 0.05 µM to 10 µM). As shown in Figure 1A,B, the kinase inhibitors labeled a9 and d10 showed potent inhibition of viability in PANC-1. Upon testing in the BxPC-3 cell line, a similar inhibition was observed for the inhibitors a9 and d10; see Figure 1C,D. The kinase inhibitor a9 from the library was identified as an ‘Aurora kinase inhibitor,’ abbreviated AT, and d10 as an ‘EGFR kinase inhibitor,’ abbreviated WZ. Both the kinase inhibitors showed over 50% inhibition in the initial screening experiments. Therefore, further in-depth studies were carried out with these two types of inhibitors.

### 3.2. Cellular Level Analysis Revealed Potent Dose-Dependent Inhibition of Viability of PANC-1 and BxPC-3 Cells by the Kinase Inhibitors AT and WZ

**Figure 2:**
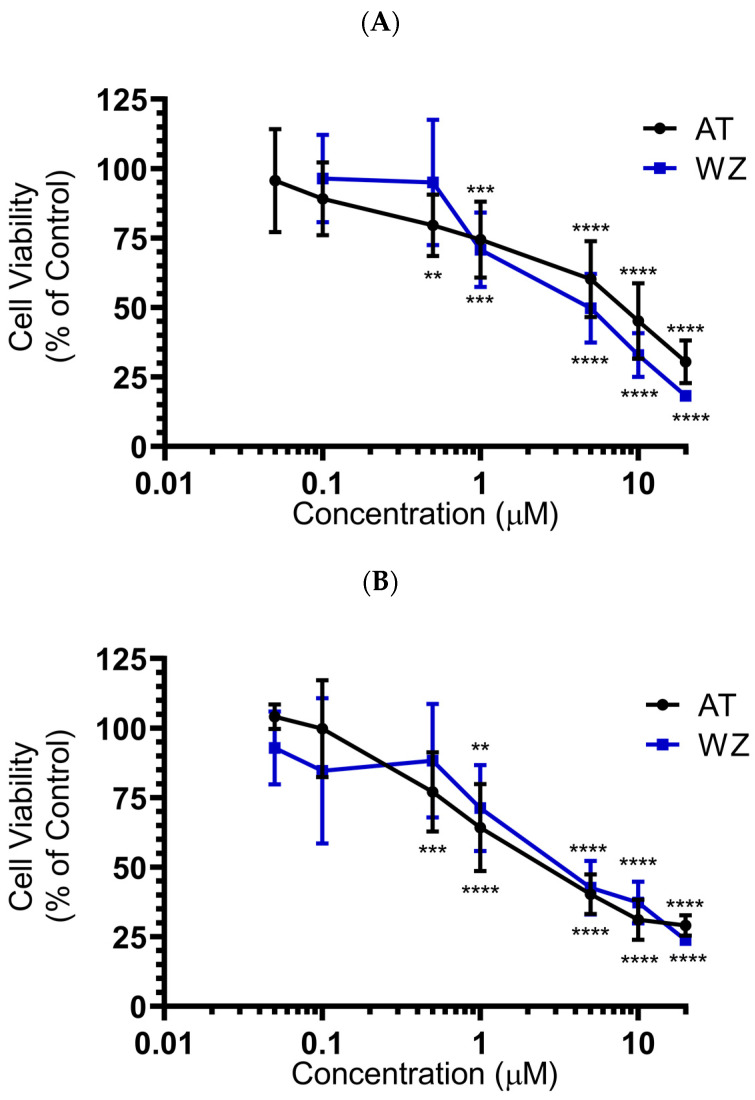
Impact of AT and WZ on PANC-1’s and BxPC-3’s cell viability. (**A**) PANC-1 cells were treated with various concentrations of AT and WZ, and their effect on the cell viability was tested as described in ‘Section 2’. Cell viability was expressed as a percentage of control cells. The experiments were performed in triplicates, and the mean of three independent experiments was calculated. A significant decrease in cell viability was observed, with *p*-values of ** = 0.0021, *** = 0.0002 and **** < 0.0001. (**B**) Similar assays were performed on BxPC-3 cells with *p*-values of ** = 0.0021, *** = 0.0002 and **** < 0.0001. Both PDAC cell lines showed potent dose-dependent inhibition of viability.

To investigate the effect of the kinase inhibitors AT and WZ on the viability of pancreatic adenocarcinoma cells and to determine the IC_50_ concentration, PANC-1 and BxPC-3 cells were treated with varying concentrations of the compound and incubated for 72 h. The effect on cell viability was assessed via CCK8 assay. A dose-dependent inhibition of cell viability was observed when the PANC-1 cells were treated with the kinase inhibitor AT. A similar dose-dependent response was observed when the cells were treated with WZ, as shown in Figure 2A, with an IC_50_ of 6.8 µM and 4.4 µM, respectively. A significant decrease in cell viability was observed and the *p*-values were ** = 0.0021, *** = 0.0002 and **** < 0.0001. When the experiment was repeated with BxPC-3 cells under identical conditions, IC_50_ values were 2.9 µM and 3.95 µM for AT and WZ, respectively, with *p*-values of ** = 0.0021,*** = 0.0002 and **** < 0.0001; see Figure 2B.

### 3.3. Effect of Kinase Inhibitors AT and WZ on ROS Levels in PANC-1 Cells

**Figure 3:**
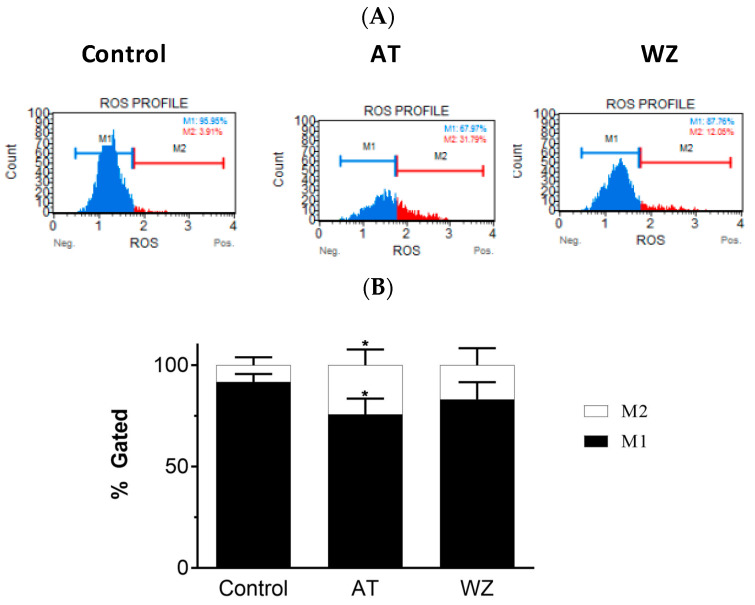
Effect of the kinase inhibitors AT and WZ on the levels of ROS (superoxide radical) in PANC-1 cells. PANC-1 cells were plated in a 12-well plate at a density of 62,500 cells/well. After 24 h, cells were treated with the AT (8 µM) and WZ (5 µM) and incubated for 72 h. Three independent experiments were performed. (**A**) Representative oxidative stress plots of control and treated group. M1 represents the ROS(−) cell population, and M2 represents the ROS(+) cell population. (**B**) The % gated profile of the control and treated group. * = *p*-value of 0.0148 for M1 cell population and 0.0248 for M2 cell population for the AT treated sample as per 2-way ANOVA with Dunnett’s multiple comparison test.

On observing the potent inhibition of cell viability by AT and WZ in PANC-1 cells, we sought to investigate their impact on pivotal cell entities. In the present study, we assessed the reactive oxygen species (ROS) by monitoring for superoxide radicals, as many anti-cancer drugs have been reported to affect the levels of ROS upon treatment. The generation of ROS was monitored using the Luminex Muse oxidative stress kit and Muse cell analyzer. We analyzed the superoxide radicals generated in PANC-1 cells upon treatment with AT and WZ. This assay allows for quantitative measurement of superoxide radical, and at the same time, determines the count and percentage of cells undergoing oxidative stress utilizing the well-characterized reagent dihydroethidium, which has been used for the detection of ROS contributed by superoxide radical in cell populations. The assay was performed by treating the cells at a concentration of 8 µM AT and 5 µM, WZ, followed by incubating the cells for 72 h in the presence of these compounds. Three independent experiments were performed. Representative plots of oxidative stress levels of control and the treated groups are shown in Figure 3A, where M1 represents the ROS(−) cell population and M2 represents the ROS(+) cell population. The % gated profile of the control, AT, and WZ-treated groups are shown in Figure 3B. The impact on ROS was observed in the treated groups. Upon normalization to the control group, a statistically significant increase in ROS positive cells was observed in the AT-treated samples, with a *p*-value of 0.0148 for M1 and 0.0248 for the M2 population, as per 2-way ANOVA with Dunnett’s multiple comparison test.

### 3.4. Kinase Inhibitors AT and WZ Invoked the Programmed Cell Death Pathway in PANC-1 Cells

**Figure 4:**
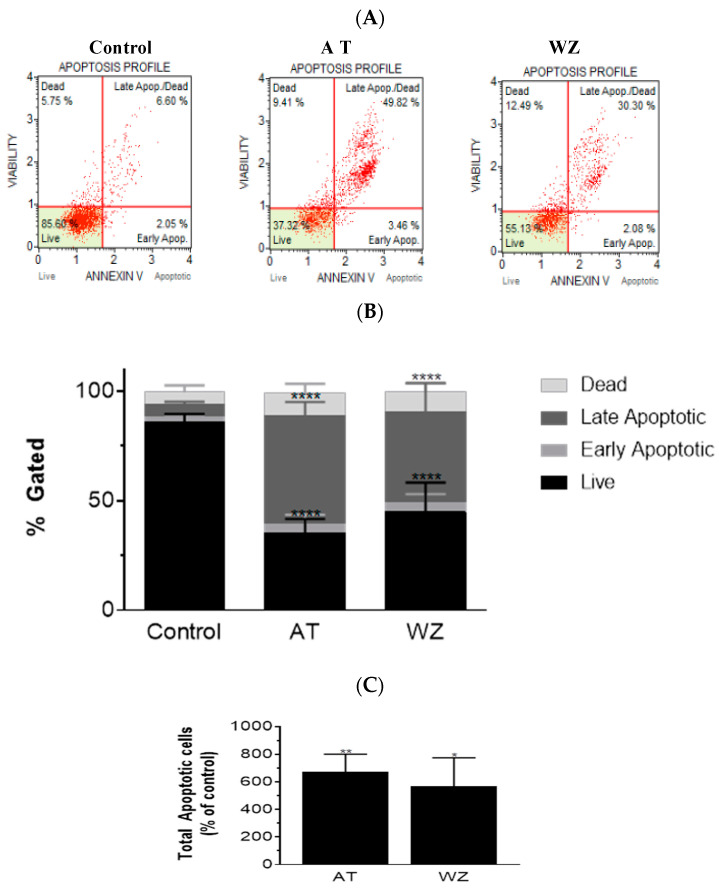
AT and WZ induce programmed cell death pathway in PANC-1 cells. PANC-1 cells were plated in a 12-well plate at a density of 62,500 cells/well. After 24 h, cells were treated individually with each of the kinase inhibitors (8 µM of AT and 5 µM of WZ) along with untreated (control) cells and incubated for 72 h. Three independent experiments were performed. (**A**) Representative Annexin flow cytometry scatter plots for the control and treated group. (**B**) % gated profile of the control and treated group with a *p*-value indicated by **** < 0.0001. (**C**) The percentage of total apoptotic cells normalized to the control is represented by a bar graph. An increase in apoptotic cells was observed in both the treated group with *p*-values indicated by ** = 0.0044 and * = 0.0115, as per ordinary 1-way ANOVA with Dunnett’s multiple comparison test.

On observing an increase in superoxide radical levels in the AT and WZ-treated cells, we proceeded to investigate whether the two kinase inhibitors invoked the programmed cell death pathway. To assess the programmed cell death pathway, the Annexin V flow cytometric assay was performed; by using this technology, the population of cells that were affected by the kinase inhibitors could be assessed. Cells were treated with 8 µM of AT and 5 µM of WZ and incubated at 37 °C for 72 h. Representative scatter plots are shown in Figure 4A. The % gated profile compared to the control is represented by a bar diagram; see Figure 4B. Upon normalization to the control group, a statistically significant increase in apoptotic cells was observed in both the AT- and the WZ-treated group; see Figure 4C.

### 3.5. Multicaspase and Executioner Caspases 3/7 Were Elicited in PANC-1 Cells by AT and WZ

**Figure 5:**
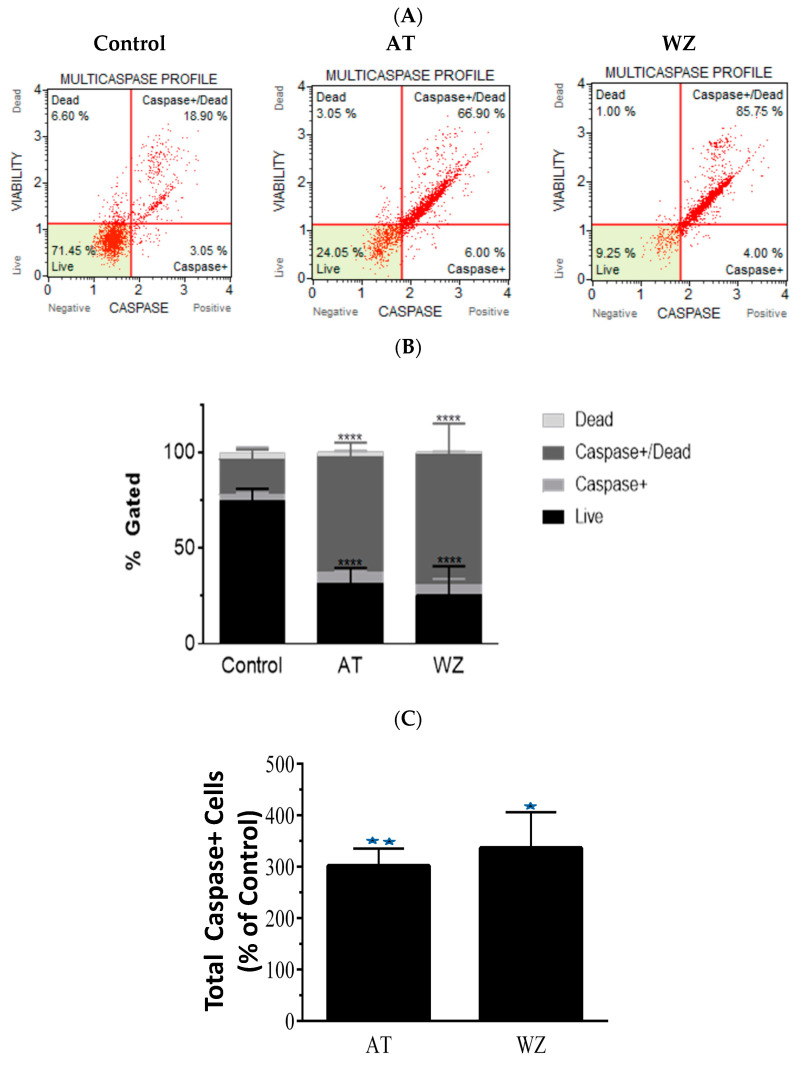

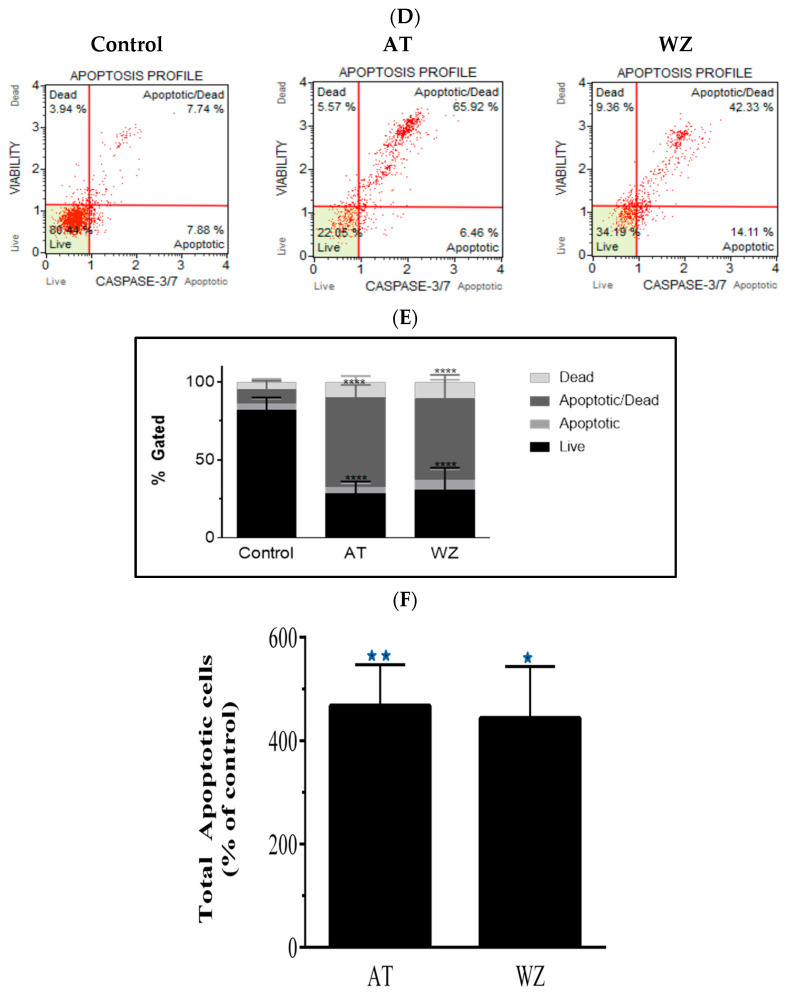
Impact of kinase inhibitors AT and WZ on multicaspase and caspases 3/7 in PANC-1 cells. PANC-1 cells were plated in a 12-well plate at a density of 62,500 cells/well. After 24 h, cells were treated with 8 µM AT and 5 µM WZ and incubated for 72 h. Three independent experiments were performed. Controls (untreated samples) were maintained in parallel. (**A**) Representative multicaspase scatter plots of control and AT- and WZ-treated groups. (**B**) % gated profile of each group analyzed with *p*-value indicated by **** < 0.0001. (**C**) The percentage of total multicaspase-positive cells after normalizing to the control is represented with a bar graph. There was a significant increase in multicaspase-positive cells, with a *p*-value indicated by ** = 0.0026 and * = 0.0011 for AT and WZ, respectively, as per ordinary 1-way ANOVA with Dunnett’s multiple comparison test. Similar experiments were conducted for the analysis of executioner caspases 3/7. PANC-1 cells were treated with 8 µM AT and 5 µM WZ individually, and also, in parallel, control cells (untreated) were incubated for 72 h. Three independent experiments were performed. (**D**) Representative caspases 3/7 scatter plots of control and treated groups are represented. (**E**) % gated profile of the samples with *p*-value indicated by **** < 0.0001. (**F**) On normalizing to the control, the increase in caspases 3/7 is represented by a bar diagram with a *p*-value indicated by ** = 0.0016 and * = 0.0023 for AT and WZ, respectively, as per ordinary 1-way ANOVA with Dunnett’s multiple comparison test.

To further investigate the impact of the kinase inhibitors AT and WZ on the involvement of multicaspase and executioner caspases, PANC-1 cells were treated with 8 µM of AT and 5 µM of WZ. Multicaspase activation was monitored using flow cytometry and multicaspase assays. Representative scatter plots of three independent trials are shown in Figure 5A. A significant increase in multicaspase positive cells was observed in the treated group compared to the control group, and as observed in the % gated profile of each of the samples in Figure 5B. On normalizing to the control, a significant increase in cells with multicaspase was observed in AT- and WZ-treated samples; see Figure 5C. Similarly, the impact of AT and WZ on executioner caspases 3/7 was monitored using flow cytometry. Representative scatter plots of three independent trials are shown in Figure 5D. An increase in executioner caspases 3/7 positive cells was also observed in the AT- and WZ-treated groups, as evidenced by the % gated profile in Figure 5E. Upon normalization to the control group, a statistically significant increase in the executioner caspases was observed, Figure 5F.

### 3.6. The Key Cell Survival Molecule Bcl-2 Was Impacted by AT and WZ

**Figure 6:**
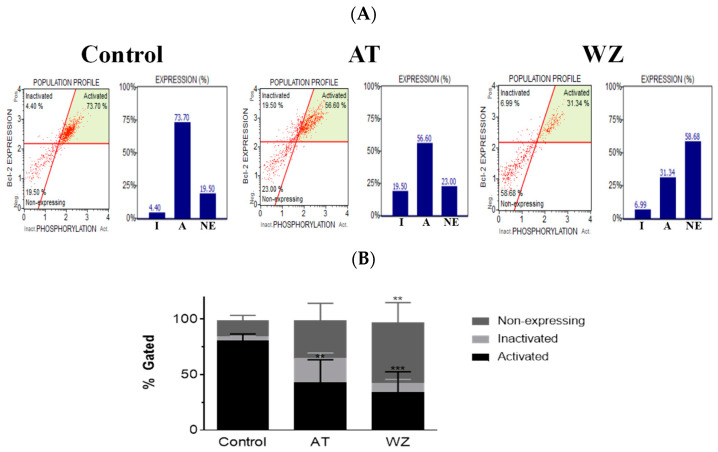
The key survival molecule Bcl-2 was impacted by treatment with AT and WZ. PANC-1 cells were plated in a 12-well plate at a density of 62,500 cells/well. After 24 h, cells were treated with 8 µM AT and 5 µM WZ and incubated for 72 h. Three independent experiments were performed. Control (untreated samples) were maintained in parallel. (**A**) Representative scatter plots of the control and AT- and WZ-treated samples, along with the percentage of activated, inactivated, and non-expressing Bcl-2 cell populations, abbreviated as A, I and NE respectively, are represented by a bar graph. (**B**) % gated profile of all the samples with *p*-values indicated by ** = 0.0037 for the activated population in AT-treated samples, *** = 0.0005 for the activated population of WZ-treated group, and ** = 0.0023 for the non-expressing population in WZ treated group, as per 2-way ANOVA with Dunnett’s multiple comparison test.

On observing the potent impact of AT and WZ on key cell pathways such as the programmed cell death pathway, we opted to explore whether the pivotal cell survival molecule Bcl-2 was impacted. To analyze the population of cells affected, flow cytometry was performed using the Luminex Muse Bcl-2 assay. Representative scatter plots of the control and AT- and WZ-treated samples, along with the percentage of activated, inactivated, and non-expressing Bcl-2 cells, are represented in Figure 6A. The means of three independent experiments with standard deviations were analyzed and normalized to the control group and represented with a bar diagram in Figure 6B. On normalizing to the control, there was a statistically significant decrease in the Bcl-2 activated population of cells in the AT-treated group. In the WZ-treated group, there was also a statistically significant decrease in the Bcl-2 activated population of cells, as well as an increase in the non-expressing cells.

### 3.7. Cell Cycle of PANC-1 Cells Was Impacted by AT and WZ

**Figure 7:**
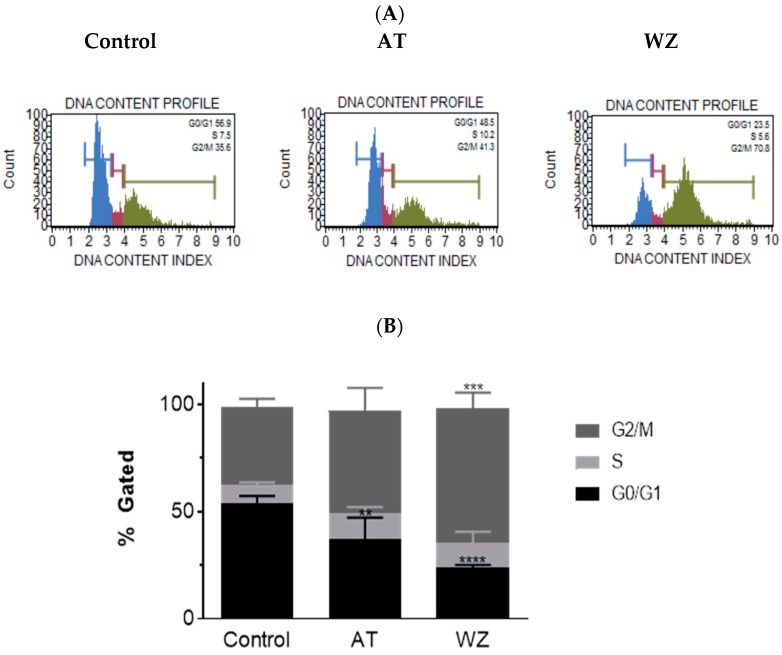
Impact on the cell cycle was observed in AT- and WZ-treated PANC-1 cells. PANC-1 cells were plated in a 12-well plate at a density of 62,500 cells/well. After 24 h, cells were treated with 8 µM AT and 5 µM WZ and incubated for 72 h. Three independent experiments were performed. Controls (untreated samples) were maintained in parallel. (**A**) Representative plots of the control and AT- and WZ-treated samples. (**B**) % gated profile of each group analyzed with *p*-value indicated by ** = 0.0067 for the G0/G1 population of AT treated sample, *** = 0.0001 for the G2/M population of WZ treated sample, and **** < 0.0001 for the G0/G1 population of WZ treated sample as per 2-way ANOVA with Dunnett’s multiple comparison test.

We further investigated whether AT and WZ had an effect on the different phases of the cell cycle of PANC-1 cells. To analyze the population of cells affected, a cell cycle analysis was performed. Representative plots of the control group and the AT- and WZ-treated groups are represented in Figure 7A. A statistically significant decrease in the G0/G1 phase of the cell cycle was observed in the AT-treated group. A decrease in cells in the G0/G1 phase, as well as an increase in the G2/M phase, was observed in the WZ-treated group, as shown in Figure 7B.

### 3.8. Proteomic Analysis Reveals Entities of Pivotal Signaling Pathways That Were Differentially Regulated in PANC-1 Cells on Treatment with AT and WZ

**Figure 8:**
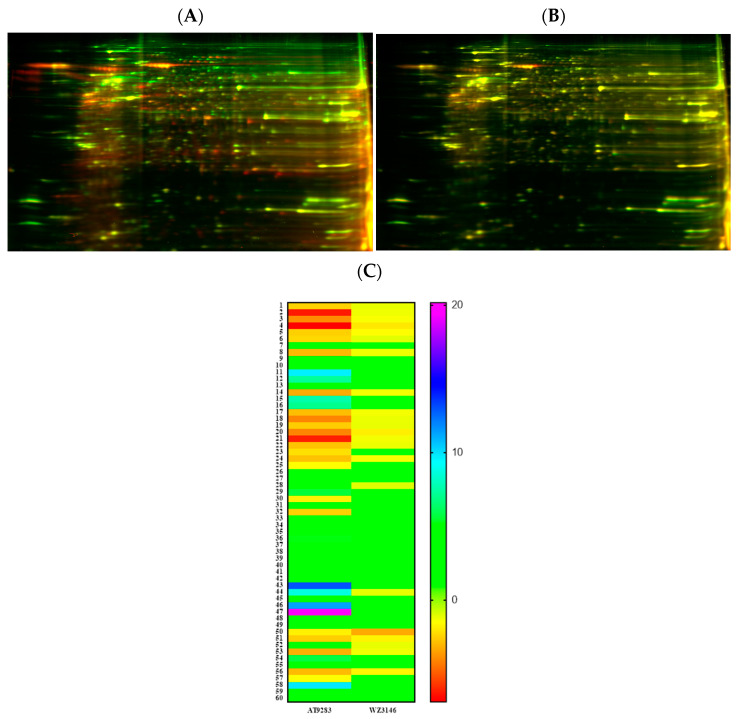
Proteomic analysis of PANC-1 cells on treatment with AT and WZ by 2D DIGE. Control (untreated) PANC-1 cells and AT and WZ treated samples (8 µM AT and 5 µM WZ) were processed to extract proteins as described in Section 2.4. Cell lysates of the control group were combined with Cy2 dye and the treated group with Cy3 dye. Overlays of the control on treated gels on isoelectric focusing are shown. (**A**) Representative overlay of the 2D gel image of the control and AT-treated sample. (**B**) Control and WZ-treated sample. (**C**) Heatmaps showing the fold change in the expression of the proteins in the AT-treated and WZ-treated samples. The numbers of each row correspond to the spot number of the gel.

To understand the mechanism of action and further delineate the key entities that could be impacted by AT and WZ, we employed sensitive methodologies such as 2D gel electrophoresis and mass spectrometric analysis to perform ‘Protein profiling’. Two-dimensional gel electrophoresis of AT- and WZ-treated groups and the control group was performed (as described in Section 2.4). The cell lysate of the control group was combined with Cy2 dye, and the treated group was combined with Cy3 dye. An overlay of the gels exhibited differentially regulated proteins. The overlay of the control gel on the AT-treated gel is shown in Figure 8A and the overlay of the WZ-treated gel and control gel is shown in Figure 8B. The heatmaps of AT- and WZ-treated groups are shown in Figure 8C.

Based on the fold changes obtained, we selected upregulated and downregulated proteins for further proteomic analysis using mass spectrometry, as listed in Table 1.

**Figure 9:**
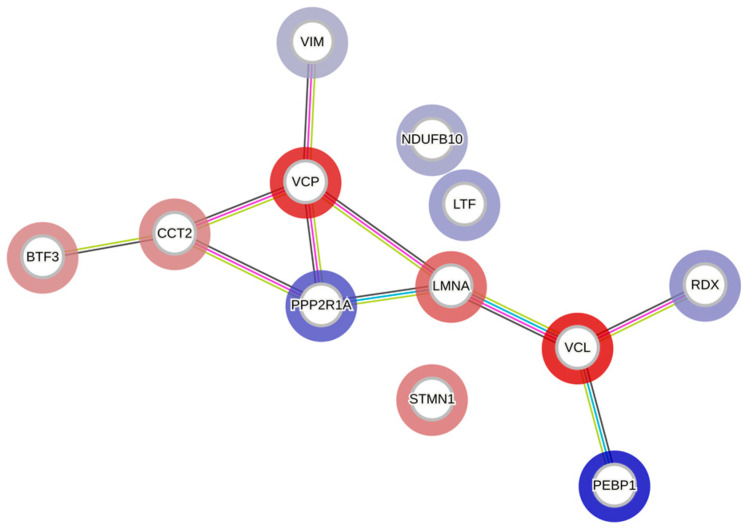
A protein–protein interaction network generated using the STRING database. Each node represents a differentially regulated protein from the AT-treated group. The blue color indicates upregulation, and the red indicates downregulation. The edges represent either known or predicted interactions, with a minimum required interaction score of 0.4 in the STRING database. The abbreviations in the above figure are mentioned in Table 1.

The STRING database was utilized to conduct functional enrichment analysis and to create protein–protein interaction networks, as shown in Figure 9. The analysis revealed the enrichment of the KEGG pathway ‘MicroRNAs in cancer’ and the reactome pathways ‘Initiation of Nuclear Envelope (NE) Reformation’, ‘Signaling by BRAF and RAF fusions’, and ‘Diseases of signal transduction by growth factor receptors and second messengers’, with false discovery rates of 0.0396, 0.0353, 0.0094, and 0.0051, respectively. Results from similar studies with WZ are presented in Supplementary Figure S1.

## 4. Discussion

The aim of this study was to discover potential pivotal molecules and treatment options for pancreatic cancer, an aggressive form of cancer with an unmet need for efficacious drugs. Screening a library of kinase inhibitors led us to two potent inhibitors: the Aurora kinase inhibitor 9283 and the EGFR Kinase inhibitor 3146, which exhibited inhibition of cell viability and potency at a low concentration in pancreatic cancer cells. PANC-1, unlike BxPC-3, harbors KRAS mutation and is described as a ‘Quasimesenchymal (QM) pancreatic ductal epithelial cell line’ [26]; in addition, QM PDAC cells are reported to contribute to differential response to therapy [31]. Additionally, in PANC-1, the control of the generation of stem cells has been reported [32]. Therefore, we opted to perform an in-depth multilevel analysis of the impact of AT and WZ on PANC-1 cells. At the cellular level, we observed potent inhibition of the viability of PANC-1 cells. For the elucidation of the molecular mechanism of action of AT and WZ, we performed flow cytometry. Using this technology, we could assess the population of cells that were impacted in various regulatory pathways. The impact on superoxide radical formation was observed in the AT-treated groups. It is reported that Aurora kinase B inhibition led to ROS production, which was required for the cell cycle arrest in the G1 phase [33]. We observed ROS production and a smaller number of cells in the G0/G1 phase when compared to the control cells on treatment with the Aurora kinase inhibitor, AT, in the present study. Both the kinase inhibitors invoked the programmed cell death pathway, leading to an increase in apoptotic, multicaspase, and the executioner caspases 3/7-positive cells. Furthermore, the key cell survival molecule Bcl-2 was impacted by both inhibitors. Moreover, both inhibitors affected the cell cycle phases of PANC-1 cells. Importantly, we performed highly sensitive assays such as 2D gel electrophoresis and mass spectrometry, which are more sensitive than Western blots and ELISA and can detect molecules in the femtomolar range [34]. On treatment with AT and WZ, various upregulated and downregulated entities were identified. One of the upregulated proteins was lactotransferrin, also termed lactoferrin (LF). LF has been reported to inhibit the proliferation/migration of cancer cells [35], induce apoptosis [36], and inhibit growth in vitro [37] and retard tumor growth in vivo [38] and is considered a ‘Miracle molecule’ [39]. The impact of LF on the human genome and its effects has been discussed recently [40]. The action of LF has been described at various fronts, including apoptosis via the Bcl-2 pathway [41]. Additionally, there are reports of the action of LF on caspases [42], including cell cycle arrest in the G0/G1 phase [37]. In fact, in the molecular-level analysis in our present study, we observed an increase in apoptotic, multicaspase, and caspase 3/7 cells, as well as a decrease in the Bcl-2 levels and also impact on the phases of the cell cycle of PANC-1 cells. An increase in LF observed on proteomic profiling could be one of the contributing factors to these observations. Additionally, the action of LF on the immune system is considered as a major contributor to its anticancer activity in the in vivo systems [43,44]. Moreover, LF has been reported to be effective when delivered by novel strategies such as the liposomalization of LF, which enhanced antitumoral effects in melanoma, and thermally responsive elastin-like polypeptide fused to an LF-derived peptide in pancreatic cancer cells [45,46]. Importantly, oncolytic peptide LTX-315 derived from bovine LF has been reported to induce anti-pancreatic cancer immunity via the ATP11BPD-L1 axis and downregulates PD-L1 [47], as well as immunity, via the natural killer (NK) cells in breast cancer [48]. Therefore, the increase in LF induced by both the kinase inhibitors in PANC-1 cells that we observed in the present study could be harnessed to sensitize and implement synergistic novel treatments with PD-L1 antibody with improved efficacy of immunotherapy for pancreatic cancers.

It is reported that a family of proteins, in which radixin is a key moiety, is involved in crosslinking the transmembrane protein PD-L1 to the actin cytoskeleton [49]. The evaluation of the expression levels of PD-L1 in various pancreatic cancer cell lines has shown weak to moderate levels of expression [50]. Interestingly, radixin is reported to possibly act as a scaffold protein responsible for cell surface localization of PD-L1 in a pancreatic cancer cell line [51]. In the present study, the kinase inhibitors AT and WZ upregulated the levels of radixin. We envision that this could lead to an increase in PD-L1, which could then be targeted by PD-L1 antibodies. Although radixin is reported to play a role in pancreatic cancer progression [52], its essential role in regulating the transmembrane molecule PD-L1 could lead to success in targeting pancreatic cancer by immunotherapy with PD-L1 antibody in the presence of kinase inhibitors such as AT or WZ, which increase radixin. Thus, apart from the increase in LF observed in the present study, which could sensitize PDAC cells to checkpoint inhibitors, an increase in radixin could also possibly aid in increasing immunotherapy by immune checkpoint inhibitors of PD-L1 in pancreatic cancer cells.

Among the protein phosphatases, serine/threonine-protein phosphatase 2A (PP2A) was one of the molecules upregulated by AT and WZ. Serine/threonine protein phosphatases play a major role in controlling various pivotal pathways. Additionally, phosphatases such as PP2C are reported to be tumor suppressors [53]. Further, small molecules activating phosphatases and reducing tumor cell invasion have been reported [54]. Notably, a molecule termed FTY720 is reported to activate phosphatase PP2A and lead to apoptosis and cell cycle arrest in lymphoma cells [55]. In the molecular mode of action study, we observed an increase in apoptotic cells, as well as an effect on phases of the cell cycle. Thus, an increase in the levels of PP2A could be one of the factors involved in this process in the PANC-1 cells.

Vimentin is expressed in mesenchymal tissue and is a marker of mesenchymal differentiation, but occasionally, epithelial cells that acquire mesenchymal phenotype express vimentin [56]. Studies carried out on patients with pancreatic cancer have shown poor survival in the presence of vimentin. Interestingly, a subset of patients exhibit autoantibodies to isoforms of vimentin, and their detection has been utilized for early diagnosis of pancreatic cancer [57]. One of the entities upregulated by AT and WZ in the present study was vimentin. Therefore, antibodies against vimentin could be harnessed for early diagnosis and also as treatment with vimentin–drug conjugates in combination with the kinase inhibitors AT and WZ.

Phosphatidylethanolamine-binding protein 1 (PEBP1), also termed RKIP, was another protein upregulated by both the inhibitors in the present study. PEBP1 has been reported in cancers of various tissue origins [58]. Reduced expression of RKIP is shown to correlate with poor prognosis in pancreatic cancer [59]. Further, the loss of RKIP leads to phenotypes with aggressive nature in PDAC [60]. Furthermore, this molecule has been reported as a metastasis suppressor in pancreatic cancer [61]. Thus, the upregulation of this pivotal molecule by AT and WZ could lead to a beneficial effect in reducing metastasis if used as a therapeutic agent for pancreatic cancers.

For cancers in general, and pancreatic cancers in particular, resistance to chemotherapeutic drugs poses a major problem in efficiently treating the disease [62]. To overcome this hurdle, cosensitizing agents are considered as options. Clinical trials have been conducted with cisplatin, gemcitabine, and other agents in patients with PDAC [63]. Moreover, the NADH dehydrogenase complex, which is one of the key enzymes involved in oxidative phosphorylation and cellular respiration and a key regulator of NAD+/NADH ratio, impacts sensitivity to platinum drugs. Notably, it has been reported that the knockout of NADH dehydrogenase complex subunit genes results in decreased sensitivity to oxaliplatin in pancreatic cells [64]. In the present study, an increase in the NADH dehydrogenase complex subunit was observed. The increase in this pivotal entity could be harnessed to increase the sensitivity of platinum drugs against pancreatic cancers if used in combination with the kinase inhibitors AT and WZ.

One of the downregulated entities by AT and WZ was the key molecule, transitional endoplasmic reticulum ATPase. The transitional endoplasmic reticulum ATPase (p97) plays a major role in various pathological processes. Notably, p97, an antiapoptotic molecule, leads to metastasis to lymph nodes resulting in a poor prognosis of pancreatic cancer [65]. The downregulation of this key moiety by AT and WZ in PANC-1 cells could be enormously beneficial as it would reduce metastasis and also lead to apoptosis. In PANC-1 cells, in the present study, we did observe apoptosis in the studies of the molecular mode of action with AT and WZ.

Vinculin was another molecule that was downregulated in the current study. An increase in vinculin expression leads to poor prognosis in pancreatic cancer patients and is considered an unfavorable prognostic indicator [66]. A decrease in vinculin by AT and WZ could lead to better outcomes in the survival of pancreatic cancer patients.

Prelamin A/C is processed into lamin A and is one of the mechano-regulating molecules in pancreatic cancers. An increase in this molecule leads to stiffness and invasiveness [67]. The downregulation of prelamin A/C by AT and WZ could prevent pancreatic cells from becoming invasive and reduce the aggressiveness of this lethal cancer.

T-complex protein 1 (TCP1) is a molecular chaperone. In cancers such as breast cancer and small cell lung cancer, it is reported that the upregulation of its expression is correlated with chemoresistance and metastasis [68]. In the present protein profiling study, we observed the downregulation of this entity in PANC-1 cells, and we envision that the downregulation of TCP1 could reduce metastasis as well as chemoresistance of PDAC upon treatment with AT and WZ.

Basic transcription factor (BTF3) was also one of the factors reduced by AT and WZ. As this transcription factor is involved in the expression of various cancer-associated genes, its decrease in AT- and WZ treated cells could lead to the decrease in cell viability that we observed at the cellular level analysis. In fact, the silencing of this entity is reported to lead to a decrease in cancer-associated genes in pancreatic cancer [69].

Stathmin was another one of the molecules downregulated by AT and WZ. Stathmin belongs to the microtubule dynamic destabilizing protein family. It is overexpressed in pancreatic cancer. It is reported that the knockdown of stathmin leads to a decrease in cell viability, colony-forming ability, and cell cycle arrest in the G2/M phase [70]. In fact, we observed a decrease in cell viability in the cellular-level analysis in the present study. Furthermore, in the analysis of the molecular mode of action, we observed cell cycle arrest in the G2/M phase in WT-treated samples.

**Figure 10:**
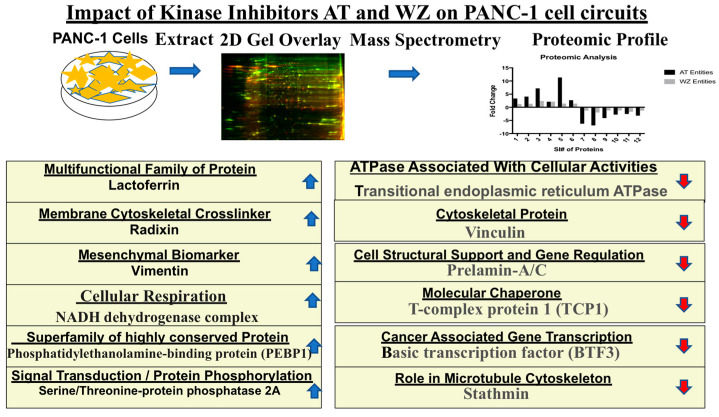
Schematic representation of cellular pathways and key entities impacted by the kinase inhibitors. Upregulated (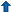) and downregulated (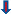) entities in PANC-1 pancreatic cancer cells upon treatment with AT and WZ.

The details of the cell signaling pathways and the entities impacted by AT and WZ are represented in the schematic diagram in Figure 10.

## 5. Conclusions

Multilevel analysis on treatment of PANC-1 cells with the Aurora kinase inhibitor (AT) and the EGFR kinase inhibitor (WZ), has highlighted pivotal entities that are impacted that belong to key cell signaling pathways. Notably, treatment with these kinase inhibitors could be harnessed in future studies for the efficacious treatment of pancreatic cancer as monotherapy due to their deleterious impact on cell-survival-related signaling pathways such as apoptosis, cell cycle, ROS, and Bcl-2 and also upregulation of entities that inhibit metastasis, decrease chemoresistance, increase immunogenic cell death, and inhibit cancer gene transcription. Moreover, proteomics analysis has revealed novel targets that can be exploited to design combination therapy with kinase inhibitors. Notably, an increase in LF could sensitize and also lead to better efficacious outcomes if synergized with immunotherapy against PD-1/PD-L1. Moreover, vimentin antibody–drug conjugates and increased efficacy with platinum drugs (because of the increase in NADH dehydrogenase complex) in combination with AT and WZ could be pursued. Therefore, future studies for the treatment of pancreatic cancer with AT and WZ are warranted.

## Figures and Tables

**Table 1:** Differentially Regulated Entities by Aurora Kinase Inhibitor and EGFR Kinase Inhibitor in PANC-1 Cells.

SI #	Spot #	Fold Change in		Key Entities Impacted by Aurora Kinase Inhibitor (AT) and EGFR Kinase Inhibitor (WZ)
		AT	WZ	
1	7	+3.35	+1.25	Lactotransferrin or lactoferrin (LTF)
2	9	+4.07	+1.35	Radixin (RDX)
3	12	+7.2	+2.4	Serine/threonine-protein phosphatase 2A (PPP2R1A)
4	26	+2.12	+2.14	Vimentin (VIM)
5	46	+11.34	+1.4	Phosphatidylethanolamine-binding protein 1(PEBP1)
6	48	+2.69	+1.39	NADH dehydrogenase (Ubiquinone)1 beta subcomplex subunit 10 (NDUFB10)
7	2	−6.26	−1.26	Transitional endoplasmic reticulum ATPase (VCP)
8	4	−6.9	−1.98	Vinculin (VCL)
9	18	−4.11	−1.2	Prelamin-A/C (LMNA)
10	22	−2.79	−1.19	T-complex protein 1 (TCP1 subunit beta) (CCT2)
11	51	−2.55	−1.74	Basic transcription factor (BTF3)
12	53	−3.17	−1.29	Stathmin (STMN1)

+ values represent upregulated and − values are downregulated entities in AT- and WZ-treated PANC-1 cells. Abbreviations mentioned in the table are also mentioned in Figure 9.

## Data Availability

All the data relating to this article are presented in the manuscript.

## Supplementary Materials

The following supporting information can be downloaded at https://www.mdpi.com/article/10.3390/biomedicines11061716/s1, Figure S1: A protein–protein interaction network generated using the STRING database. Each node represents a differentially regulated protein from the WZ-treated group. The blue color indicates upregulation, and the red indicates downregulation. The edges represent either known or predicted interactions, with a minimum required interaction score of 0.4 in the STRING database. The abbreviations in the above figure are mentioned in Table 1.
