# Supplementary material for: Multifaceted Effects of Kinase Inhibitors on Pancreatic Cancer Cells Reveals Pivotal Entities with Therapeutic Implications
Source: Biomedicines. 2023 Jun 15;11(6):1716. doi: 10.3390/biomedicines11061716 (PMC10296261; doi:10.3390/biomedicines11061716)
Supplement: Supplementary file 1 [file biomedicines-11-01716-s001.zip › biomedicines-2430549-supplementary.pdf]

Supplementary Materials:

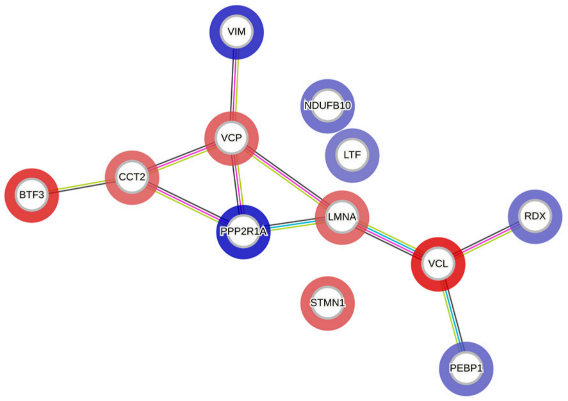

Supplementary Figure S1. A protein–protein interaction network generated using the STRING database. Each node represents a differentially regulated protein from the WZ-treated group. The blue color indicates upregulation, and the red indicates downregulation. The edges represent either known or predicted interactions, with a minimum required interaction score of 0.4 in the STRING database. The abbreviations in the above figure are mentioned in Table 1.
